# A Rare Case of Profound Sinus Bradycardia in a Patient With Descending Aortic Dissection

**DOI:** 10.7759/cureus.49291

**Published:** 2023-11-23

**Authors:** Izaak Fenech, Anthea Ferriggi, Mark Abela

**Affiliations:** 1 Cardiology, Mater Dei Hospital, Msida, MLT

**Keywords:** severe hypertension, tevar, left ventricular hypertrophy, mimicking st elevation, sinus bradycardia, type b aortic dissection

## Abstract

A 34-year-old uncontrolled hypertensive male presented with chest pain radiating to the back. Despite severe pain, he was persistently bradycardic at 38 beats per minute. The workup at the emergency department confirmed the presence of an acute Stanford B aortic dissection. Stanford B dissections are not usually associated with bradycardia. It is Stanford A dissections that are mostly linked with bradycardia because Stanford A dissections can cause concomitant coronary artery extension and involvement of the atrioventricular node. This case demonstrates that sinus bradycardia can exist in the acute setting of any painful aortic dissection, even though it might not necessarily involve the coronary arteries.

## Introduction

Aortic dissections often present with severe, crushing chest pain radiating to the back. Damage to the inner aortic wall creates a false lumen between layers, separating the tunica intima and media from the tunica adventitia [1]. End-organ ischemia (e.g., stroke, renal or adrenal infarct) is one of the manifestations of aortic dissection [2]. Other complications include cardiac tamponade and aortic regurgitation. Death can occur instantaneously from aortic rupture or coronary artery extension leading to myocardial ischemia. If left untreated, aortic dissections can carry a mortality rate of up to 50% [3]. Risk factors for aortic dissection include uncontrolled systemic hypertension, atherosclerosis, collagen disorders like Marfan syndrome and Ehlers-Danlos syndrome, aortic coarctation, bicuspid aortic valve, and cocaine use [4]. High-speed motor vehicle accidents can also cause aortic dissection.

Methods to classify aortic dissection include the Stanford system and the DeBakey system. The Stanford system classifies dissections according to the involvement of the ascending aorta. Type A dissections involve the ascending aorta, while type B dissections do not involve the ascending aorta. This distinction is important for management purposes in terms of the urgency of surgical intervention. The DeBakey classification of aortic dissection focuses on the anatomy of the aorta, classifying dissections based on the location of the intimal tear as follows. In Type I, the intimal tear is located in the ascending aorta, extending at least to the aortic arch and occasionally beyond, distal to the aortic arch. In Type II, the intimal tear is located within the ascending aorta only. In Type III, the intimal tear is within the descending aorta.

Both classifications are sometimes used to describe dissections. In our case, the patient had a Type III B Aortic dissection.

Severe acute pain causes a surge in catecholamines, which, among other physiological responses, cause tachycardia. Witnessing sinus bradycardia of 38 beats per minute in a patient claiming the worst-ever 10/10 chest pain severity was anomalous. This case report aims to provide a physiological explanation for the bradycardia in the context of a very painful Stanford B descending aortic dissection.

## Case presentation

**Figure 1:**
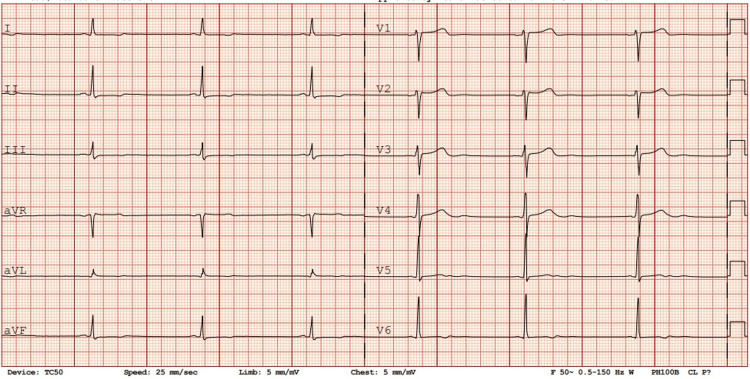
ECG on admission. Normal sinus rhythm: 38 beats per minute. ST segment elevations v1 v2.

**Figure 2:**
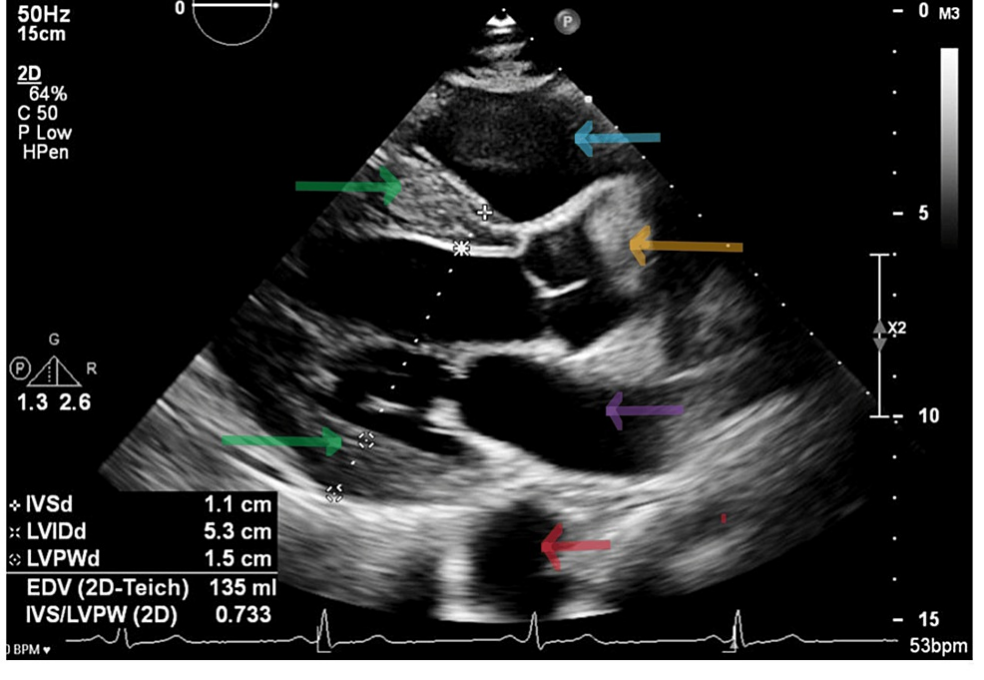
Parasternal long axis. The green arrows show mild concentric left ventricular hypertrophy. The left ventricle is not dilated. The blue arrow shows the left atrium. The orange arrow shows the proximal aorta. The purple arrow shows the left atrium. The red arrow shows the descending aorta.

A 34-year-old male with uncontrolled familial hypertension presented to a general practice health facility with severe chest pain. A point-of-care ECG was carried out, which showed mild ST-segment elevation in leads V1 and V2. The initial impression was that of ST-segment elevation myocardial infarction, and thus the patient was promptly referred to the Accident and Emergency (A&E) Department. Upon arrival at the emergency department, the patient described an abrupt onset of central chest pain, excruciating and tearing in nature, which radiated to the back. The pain severity was quantified as 10/10. He was diaphoretic and tachypneic at around 30 respirations per minute. On examination, the BP was 220/130 mmHg, there was no radio-radial delay and no significant right and left arm BP difference, but there was a radio-femoral delay. A repeat ECG (Figure 1) was performed showing sinus bradycardia at 38 bpm with persistent mild ST-segment elevations in V1 and V2. The patient was not on any medications. A point-of-care echo (Figure 2) was performed, which showed no regional wall motion abnormalities; however, mild left ventricular hypertrophy was noted. 

**Figure 3:**
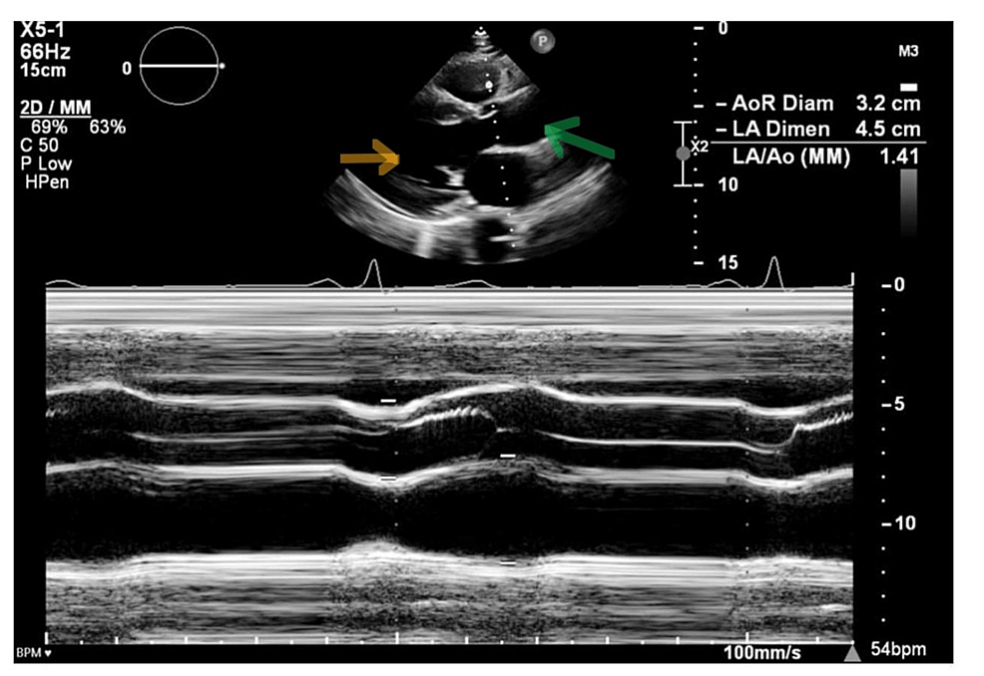
The ascending aorta (green arrow) is not dilated and not dissected. The mitral valve is normal. The aortic valve shows trace aortic regurgitation. No pericardial effusion. Orange arrow: left ventricle.

**Figure 4:**
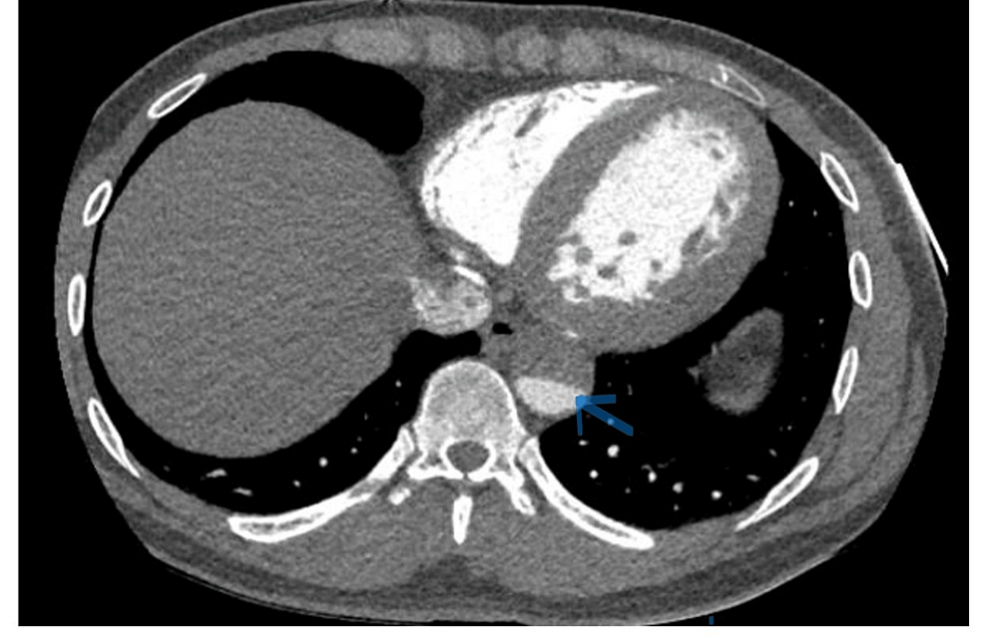
CT thoracic aorta on admission showing descending aortic dissection. True lumen (blue arrow).

Given the suggestive history, despite the echo showing no evidence of ascending aortic dissection (Figure 3), the patient underwent an urgent CT thoracic aorta scan. This revealed a descending aortic dissection labeled as type IIIB in the DeBakey classification (Figure 4), corresponding to a Stanford B dissection. The dissection extended from the origin of the left subclavian artery to the abdominal aorta, including the truncus coeliacus. The common iliac arteries, right external iliac artery, and left external and internal iliac arteries were also involved. The patient was then transferred to intensive care for continuous monitoring, pain relief, and isosorbide dinitrate infusion to lower blood pressure. Apart from the aortic dissection and left ventricular hypertrophy, there were no other signs of end-organ damage. During the first three days of admission, the resting heart rate did not exceed 45 beats per minute and dropped as low as 33 beats per minute.

**Figure 5:**
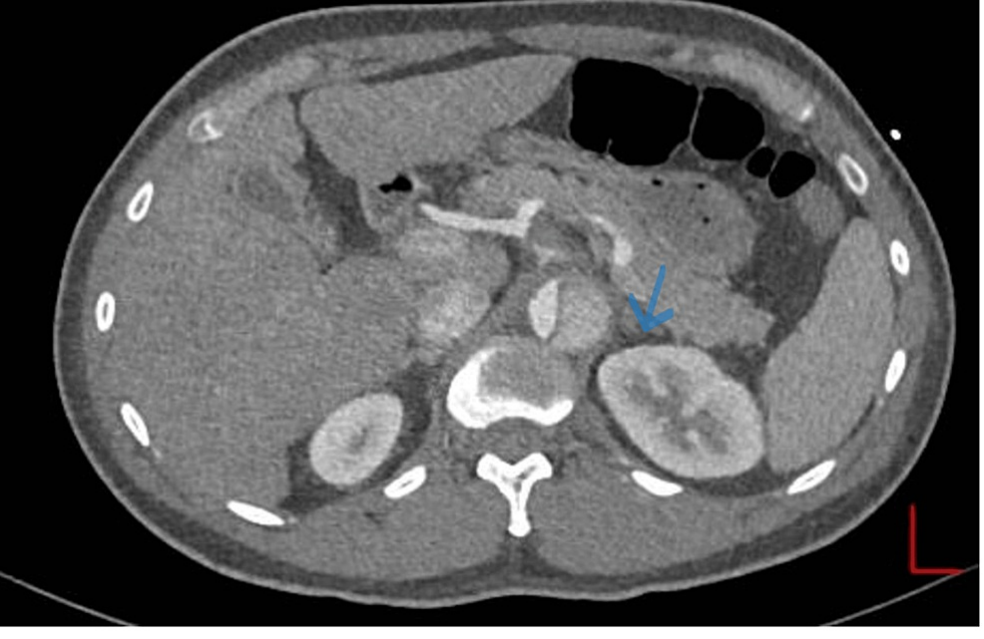
CT thoracic and abdominal aorta was performed two days later. Exhibiting a lack of enhancement of the adrenal gland in keeping with ischemia of the left adrenal gland (blue arrow).

The repeat CT (Figure 5), showed an increase in the caliber of the false lumen at the level of the infrarenal abdominal aorta. A lack of enhancement of the left adrenal gland was also noted, raising the concern of ischemia. 

**Figure 6:**
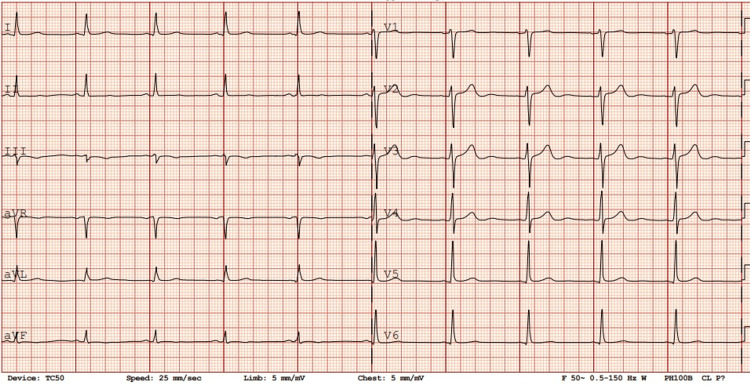
ECG taken 15 days after presentation, which on discharge shows the normal sinus rhythm at 63 beats per minute.

The presence of ischemia in the adrenal gland prompted a more aggressive surgical approach by the vascular surgeons, who performed thoracic endovascular aortic repair (TEVAR). The surgery was successful, and eventually, the patient was discharged on antihypertensive medication. The total inpatient stay lasted 15 days. Upon discharge, the resting heart rate had recovered to 63 beats per minute and the patient was pain-free (Figure 6).

## Discussion

Bradycardia is not unknown in aortic dissections. However, it is usually linked with ascending Stanford Type A aortic dissections rather than descending Stanford Type B dissections. The mechanism by which Type B aortic dissection can present with bradycardia is unclear; however, the carotid sinus is thought to play a role. Carotid body pressure can yield bradycardia, hypotension, or both [5].

According to [6], chest pain is present in 85% of Type A dissections versus 67% of Type B dissections. Type B was more associated with back pain than Type A dissections, 70% vs 43% respectively. Syncope is much more likely to be associated with Type A dissection (19%) than Type B dissection (3%). As for hypertension, 66% of Type B dissections were hypertensive on presentation, while only 28% of Type A dissections were hypertensive on presentation. The profound bradycardia in this case is quite atypical, as the usual physiological response to pain is tachycardia. Pain directly stimulates the rostral ventrolateral medulla, which produces elevations in sympathetic activity, resulting in an increase of catecholamine levels which in turn lead to tachycardia and hypertension [7]. Studies published to date highlight that bradycardia associated with aortic dissections is usually the result of (a) stimulation of the aortic depressor nerves, (b) stimulation of a hematoma in the intramural septum close to the AV node, and (c) stimulation of the carotid sinus.

Stimulation of the aortic depressor nerves 

Stimulation of the aortic depressor nerves causes bradycardia by eliciting a vagal activation. This response is usually accompanied by a decrease in blood pressure [8]. In our case, the patient had a severely elevated systolic blood pressure of 220 mmHg, rendering such a mechanism behind the bradycardia unlikely, though still possible, as the patient was chronically hypertensive and thus the blood pressure could have been resistant to vagal effects.

Stimulation of a hematoma in the intramural septum close to the AV node

Hematoma or ischemia involving the atrioventricular (AV) node could cause conduction problems resulting in bradyarrhythmias. In our case, a hematoma causing AV block could not have been the cause of the bradycardia, as the dissection did not extend into the ascending aorta, let alone the coronaries and the AV node. Despite ST elevations which initially caused concern for coronary involvement, the lack of regional wall motion abnormalities on echocardiography and repeated negative troponin values ruled out myocardial infarction. The ST segment changes were attributed to the presence of left ventricular hypertrophy in a chronically uncontrolled hypertensive.

Stimulation of the carotid sinus 

A potential feasible explanation for the paradoxical bradycardia in this painful clinical presentation is the involvement of the arterial supply of the carotid sinus, which in turn affects the carotid sinus baroreceptor response [9,10]. The carotid sinus baroreceptor functions as a sensor responding to the mechanical stretch that occurs in the carotid artery as arterial blood pressure increases. When the blood supply to the carotid sinus is altered, the carotid sinus responds by releasing more serotonin. This can result in an increased response of peripheral receptors or an abnormal response of central reflex sites. Carotid sinus receptors are also sensitive to stretching of the arterial wall. Upon stretching, via branches of the glossopharyngeal nerve, an impulse is sent to the nucleus tractus solitarii in the medulla oblongata. The efferent limb of the reflex is carried via the vagal nerve and cervical sympathetic nerves from the medulla oblongata to the heart, lowering the heart rate. In such individuals, stimulation of carotid sinus baroreceptors tends to result in a more significant than expected decrease in heart rate, giving a bradycardic presentation [11].

An aortic dissection can potentially cause disruption in the signal transduction of blood pressure homeostasis [12]. The stress that the aortic wall experiences during an acute dissection can stimulate the mechano-sensory baroreceptors in the outer layer of the aortic arch, which in turn, via stretch of the carotid sinus, initiates the baroreceptor response that leads to bradycardia. Despite extensive research, the exact mechanism of how an aortic dissection alters baroreceptor function remains elusive.

Management of aortic dissection varies substantially based on the Stanford classification. Most descending aortic dissections are managed medically with adequate control of hypertension via pharmacological agents [13], representing the uncomplicated type of Stanford Type B dissections. Complicated cases involving persistent or recurrent pain, rupture, and visceral or extremity ischemia necessitate imminent intervention either through TEVAR or through open surgery. TEVAR appears to be the best strategy for managing acute complicated type B aortic dissection, with significantly better mortality rates, lower complications, and shorter hospitalization compared to open surgery [14]. In this case, clinical features supporting a non-conservative approach included the ischemic changes on repeat CT, as well as the extension of the dissection. TEVAR was performed with the goal of eliminating antegrade flow into the false lumen via stent graft insertion into the true lumen.

## Conclusions

Aortic dissection is a rare condition that carries a high mortality rate. It is essential that it is diagnosed quickly and appropriately. Misdiagnosis or delayed diagnosis can lead to devastating consequences. Given clinical features suggestive of an aortic dissection, bradycardia should not dissuade the physician away from diagnostic radiological aortic imaging. Severe pain and bradycardia are paradoxical phenomena, leading to the possibility of diagnostic confusion arising from bradycardic patients with severe pain. We hope that this case report highlights the fact that all types of aortic dissections, including dissections limited to the descending aorta, can present with profound bradycardia despite severe pain. 
